# Using Storybooks to Teach Children About Illness Transmission and Promote Adaptive Health Behavior – A Pilot Study

**DOI:** 10.3389/fpsyg.2020.00942

**Published:** 2020-06-05

**Authors:** Megan Conrad, Emily Kim, Katy-Ann Blacker, Zachary Walden, Vanessa LoBue

**Affiliations:** ^1^Center for Developmental Science, Department of Psychology, William Paterson University, Wayne, NJ, United States; ^2^Child Study Center, Department of Psychology, Rutgers University, Newark, NJ, United States

**Keywords:** contagion, germs, storybook, intervention, behavioral avoidance

## Abstract

Although there is a large and growing literature on children’s developing concepts of illness transmission, little is known about how children develop contagion knowledge before formal schooling begins and how these informal learning experiences can impact children’s health behaviors. Here, we asked two important questions: first, do children’s informal learning experiences, such as their experiences reading storybooks, regularly contain causal information about illness transmission; and second, what is the impact of this type of experience on children’s developing knowledge and behavior? In Study 1, we examined whether children’s commercial books about illness regularly contain contagion-relevant causal information. In Study 2, we ran a pilot study examining whether providing children with causal information about illness transmission in a storybook can influence their knowledge and subsequent behavior when presented with a contaminated object. The results from Study 1 suggest that very few (15%) children’s books about illness feature biological causal mechanisms for illness transmission. However, results from Study 2 suggest that storybooks containing contagion-relevant explanations about illness transmission may encourage learning and avoidance of contaminated objects. Altogether, these results provide preliminary data suggesting that future research should focus on engaging children in learning about contagion and encouraging adaptive health behaviors.

## Introduction

Researchers, clinicians, and health professionals alike have a vested interest in children’s developing understanding of illness transmission. On a theoretical level, children’s understanding of illness transmission can tell us about children’s conceptual development, as it involves reasoning about invisible organisms that cannot be seen or touched. On a clinical level, the study of illness transmission also carries important implications for children’s health. Children are especially risky carriers of disease, not only because they are more likely to catch diseases themselves but also by enabling greater transmission of infection to others (Bryant and McDonald, 2009; Lambe et al., 2012; de Lencastre and Tomasz, 2002).

The growing literature on children’s understanding of illness transmission suggests that knowledge develops in a piecemeal fashion starting in the preschool years (e.g., Kalish, 1996, 1999; Keil et al., 1999; Myant and Williams, 2005; Legare et al., 2009). For example, when given a choice between two explanations for the cause of a cold, preschool-aged children are more likely to endorse contagion-relevant explanations (e.g., from playing with a friend who had a cold) than immanent justice explanations (Siegal, 1988; Springer and Ruckel, 1992). However, when asked open-ended questions, children of the same age overextend contagion to non-contagious events, such as accidents (e.g., scraped knee) and non-contagious illnesses (e.g., toothache), and they endorse non-physical causes of illness transmission, including immanent justice (e.g., getting sick as a punishment for misbehaving) (Kister and Patterson, 1980).

Despite a large and growing literature on children’s knowledge about illness transmission, there is very little work to date that focuses on how children *behave* when faced with the threat of getting sick and how it relates to their contagion knowledge. This is important, as children’s behavior when faced with a contagious illness or a contaminated object is what is most relevant to whether they actually get sick and whether they spread illnesses to others, making children’s behavior when confronted with a contagious illness directly relevant to public health. Only one very recent study has linked children’s knowledge about illness transmission to their behavior, and the results implicate the role of causal knowledge in children’s avoidance of sick individuals and potentially contaminated objects. In this study, 4- to 7-year-olds were prompted to interact with two confederates – one that was “sick” and one that was not – and with various toys that each confederate touched. Only the 6- and 7-year-olds avoided proximity to and contact with the sick confederate and her toys. However, the best predictor of children’s avoidance behavior was not age but, instead, was their ability to make predictions about illness outcomes. In other words, even 4- and 5-year-olds avoided contact with the sick confederate if they had causal knowledge about illness transmission (Blacker and LoBue, 2016).

Although the literature suggests that children likely develop a sophisticated causal understanding of illness transmission when they begin formal schooling, this study demonstrates not only that preschool-aged children are capable of learning about illness transmission but that they might also be able to use their knowledge to guide adaptive behavior. However, most preschool-aged children have not yet acquired causal knowledge about illness transmission, thus leaving open the question of *how* preschoolers might acquire this knowledge and whether providing them with it could promote adaptive health behaviors.

Importantly, before formal schooling begins, differences in experience, language, culture, and the environment influence the development of children’s biological knowledge (e.g., Ross et al., 2003; Waxman et al., 2007; Anggoro et al., 2010; Unsworth et al., 2012). One particularly relevant source of learning for young children is storybooks. Indeed, for most children, storybook reading is a common informal learning experience: in the United States, children 8 years old and under spend, on average, 29 min per day with books (Rideout, 2017). Further, there is a growing body of work showing that preschool-aged children can learn new biological facts from reading storybooks. For example, children as young as 3 can learn factual information about animal camouflage from just one exposure to a storybook (Ganea et al., 2011; Geerdts et al., 2016a), and children as young as 5 can learn about complex biological properties such as evolution from storybook interventions (Legare et al., 2013; Kelemen et al., 2014). There is even direct evidence that storybook reading can increase 4- to 7-year-olds’ knowledge about illness transmission (Williams and Binnie, 2002) and that a storybook intervention can promote 4- and 5-year-old children’s healthy behaviors, such as choosing to eat healthier snacks (Gripshover and Markman, 2013). However, it is still unclear whether storybooks that children commonly read contain causal information about illness transmission and whether providing children with this information can promote both learning and behavioral avoidance of potentially contaminated objects.

The current research constitutes a first step toward answering these important questions. Here, we present an initial exploratory investigation of whether children’s commercial storybooks regularly contain causal information about illness transmission and whether providing children with this information can affect both their knowledge and behavior. In Study 1, we examined whether causal information about illness transmission is readily available in real-world storybooks. We reviewed a large sample of children’s library storybooks about illness and quantified the types of information they present about illness transmission. We asked whether contagion-relevant causal explanations (i.e., germs, physical proximity to sick individuals) are presented more frequently than contagion-irrelevant causal explanations (i.e., behaviors like not wearing a jacket, disobeying parents, wishing to be sick) and thus whether storybooks that are readily available to children in the real world can serve as a potential source of accurate information about illness transmission. Previous research analyzing the presence of causal biological information in children’s commercial storybooks found that very few books provided the kinds of causal information considered necessary for substantial knowledge revision (Geerdts et al., 2016b), suggesting that we may similarly find a lack of causal information about contagion.

In Study 2, we examined whether providing children with causal information about illness transmission in children’s storybooks can influence their knowledge and behavior when presented with a contaminated object. Preschool-aged children were read one of several storybooks, each of which contained a different explanation for illness transmission. We then assessed children’s knowledge about illness transmission and whether they displayed behavioral avoidance of a contaminated toy in a forced-choice task. We chose to focus on preschool-aged children (ages 3–5) because children of this age have not yet begun formal schooling, have a limited understanding of contagion (e.g., Bibace and Walsh, 1981; Perrin and Gerrity, 1981), and do not spontaneously avoid contact with potentially contaminated objects (Blacker and LoBue, 2016). Thus, preschool-aged children should perform poorly in a baseline assessment of contagion knowledge and have substantial room for learning. We hypothesized that learning a causal explanation for illness transmission from a storybook reading would lead to subsequent knowledge gain as well as avoidance of a contaminated object.

## Study 1

The goal of Study 1 was to provide a descriptive account of whether causal information about illness transmission is readily available in commercial storybooks. We reviewed a large sample of children’s storybooks about illness available at local lending libraries and coded them for the types of explanations they provide for illness transmission, if any.

### Materials and Methods

#### Storybooks

Books about illness were collected from local lending libraries that were a part of a larger consortium of libraries in Northern New Jersey. The consortium included 77 member libraries with a total of 11 million circulated items. The library consortium online catalog was used to compile a list of storybooks. Five of these libraries were then used to gather materials obtained from searching the entire catalog. We used search limiters to find English-language juvenile storybooks about illness. A keyword search was done for the keyword “sick.” We did not set limits on the book type (e.g., narrative versus non-fiction), but all of the storybooks returned in the search were narrative. Storybooks were then scanned for content to be sure that they were about being sick, were appropriate for preschool-aged children (ages 3–6 years), and were designed for a general audience (i.e., excluded religious books, books to help children get over a fear of the doctor, books about specific long-term illnesses or death). Any books that were out of circulation or missing from the library were excluded from analysis (13 books). The final sample resulted in 67 storybooks (see Supplementary Appendix A for full list of titles). Original publication dates for the books ranged from 1939 to 2014, with almost half of the books (32) published since 2000. Most of the storybooks (40) were about ambiguous illnesses that had symptoms similar to a cold or the flu (e.g., stuffy nose, fever, headache, chills), and the stories only specified that the character(s) was “sick.” The rest of the storybooks referenced specific illnesses: a cold (15), the flu (4), chicken pox (7), or pneumonia (1).

#### Coding

The text of each storybook was coded for reference to contagion-relevant and contagion-irrelevant explanations regarding illness transmission. The explanations and mechanisms were selected on the basis of prior research that investigated children’s own explanations (Kister and Patterson, 1980; Bibace and Walsh, 1981). We coded the illness stories based on the presence of an explanation for why the character(s) became sick. We included two categories of contagion-relevant explanations – *physical proximity explanations* and *biological explanations*. *Physical proximity* explanations either explicitly or implicitly mentioned physical proximity as a possible cause but provided no additional information regarding the transmission of illness within the proximity event (e.g., a story where a character took care of someone who was sick and then they themselves became sick later). *Biological explanations* explicitly mentioned germs, viruses, bacteria, or any other physical/medical origin for the illness. We also included two categories of contagion-irrelevant explanations – *behavioral explanations* and *non-physical explanations. Behavioral* explanations referenced a specific activity or behavior that led to a character becoming sick (e.g., not wearing a coat, eating too much junk food). *Non-physical* explanations linked the onset of illness to a non-physical, unseen cause, such as psychological intent (e.g., wishing to be sick to stay home from school) or immoral conduct (e.g., a child becoming sick after disobeying his/her mother). Finally, a *no explanation* category was included for books that did not provide any information regarding how a character became sick.

Each storybook was coded binomially as either having made reference or not having made reference to each possible explanation. Thus, one storybook may have received more than one code (e.g., both germs and physical proximity). Two coders (first author and undergraduate research assistant) independently coded all of the storybooks. Percent agreement between the coders was 96.2%. The average kappa was 0.84 (0.77 for physical proximity, 0.89 for biological, 0.82 for behavioral, 0.79 for non-physical, 0.94 for no explanation), which is considered a strong level of agreement. Any disagreements were resolved by discussion, and both coders agreed upon a final code.

### Results

**FIGURE 1:**
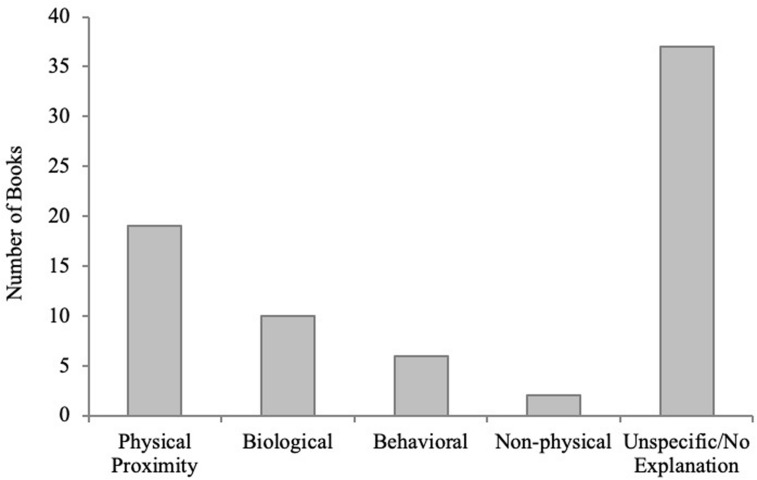
Number of storybooks that provided physical proximity, biological, behavioral, non-physical justice, and no/unspecified explanations for illness transmission in Study 1.

The number of books containing each of the explanation types is presented in Figure 1. Over half of the storybooks (*N* = 37, 55%) did not mention or imply any possible mechanisms for illness transmission and simply focused on the experience of being sick. Of the storybooks that did provide explanations about the causes of illness (*N* = 30), 23 provided contagion-relevant explanations, and 8 provided non-contagion-relevant explanations. Of the books providing contagion-relevant explanations, only 10 books specifically mentioned germs or other biological causes (e.g., an appendix causing appendicitis), while the remaining 13 only highlighted that physical proximity contributed to the transmission of illness. In many of the physical proximity explanations, a character became sick, and then the character’s caregiver soon became sick as well, leading to a role reversal in the care relationship. These stories did not mention why the particular illness was contagious or how it passed between individuals, but simply implied that physical proximity to the sick individual was a possible factor. Non-contagion-relevant explanations were much less frequent; very few stories mentioned non-physical (2) or behavioral causes (6) for illness. Neither of the two non-physical explanations were accurate: (1) wishing to be sick and (2) believing they were sick when they weren’t. Of the six behavioral explanations, three involved getting sick from eating too much food (or non-food items), one was mistaken allergies from a dandelion, one was from going out in the snow without a hat, and one was from playing a game on the playground.

Note that there were no significant differences in the number of books that provided causal explanations, or the types of explanations provided, based on publication dates of the books [books published before (*N* = 32) versus after (*N* = 35) the year 2000, *p*’s > 0.13, Fisher’s exact tests]. There were also no significant differences in the number of books that provided causal explanations, or the types of explanations provided, based on the books’ popularity [median split of Amazon.com US sales rankings with more popular (*N* = 34) versus less popular (*N* = 33), *p*’s > 0.09, Fisher’s exact tests].

### Discussion

Our findings from Study 1 demonstrate that storybooks for preschool-aged children do not often provide *any* causal information about the process of contagion. When they do provide causal information, it usually is contagion-relevant information, such as physical proximity. However, children rarely receive causal information about the biological process of contagion from storybooks; less than 15% of the books explained that germs and other biological agents are causes of illness. Incorrect and/or non-contagion-relevant information, like wishing to be sick or swallowing a basketball, was found at almost the same rate as germ explanations (12% of all the books). Overall, commercial storybooks do not seem to be a source from which children can typically gather biological causal information about illness transmission.

## Study 2

In Study 1, we provided a descriptive account of the kinds of information about illness transmission children typically receive via commercial storybooks. In Study 2, we ran a pilot study to explore whether exposure to contagion-relevant information *can* promote increased knowledge of illness transmission and, in turn, whether causal knowledge of illness transmission promotes behavioral avoidance of a potentially contaminated object. This study speaks specifically to whether it is possible to support children’s understanding of illness and adaptive health behaviors after a simple, informal learning experience like storybook reading. Using a pre-intervention–post-test design, we provided preschool-aged children with either contagion-relevant or no contagion-relevant causal information about an illness in a storybook and then assessed both their knowledge gain and their behavioral avoidance of a potentially contaminated toy. We hypothesized that learning a causal explanation for illness transmission from a storybook reading would lead to increased knowledge, and knowledge would be related to subsequent avoidance of a contaminated object.

### Materials and Methods

#### Participants

We recruited seventy-one 3- to 5-year-old children (*M* = 4.72 years, SD = 0.56 years, 38 female) from local preschools in Essex and Passaic Counties in New Jersey for testing. Parents were invited to participate via information packets sent home with their child. Parents gave written consent for their child’s participation and completed a demographic form, while children gave verbal assent. Any child whose parents returned consent forms was eligible to participate. There were no specific exclusion criteria for children’s involvement.

Children were randomly assigned to one of three contagion-relevant storybook conditions – biological storybook (*N* = 24, *M* = 4.52 years, SD = 0.52 years, 13 female), germs-only storybook (*N* = 23, *M* = 4.84 years, SD = 0.49 years, 12 female), and contact-with-toys storybook (*N* = 24, *M* = 4.82 years, SD = 0.64 years, 12 female). An additional 48 children were recruited from local preschools and were assigned to one of two control conditions: a no-book condition where participants were simply given both the pre- and post-test assessments with no book intervention (*N* = 24, *M* = 4.48 years, SD = 0.69 years, 12 female), and a no-explanation condition, in which children were read the same story, except without an explanation for the events (*N* = 24, *M* = 4.64 years, SD = 0.78 years, 12 female). We based our sample size of 20–24 children per condition on previous research using storybook manipulations. Sample sizes in the studies we reviewed prior to conducting the submitted work ranged from 16 to 24 participants per condition (Ganea et al., 2008, *N* = 16; Ganea et al., 2014, *N* = 22–24; Ganea et al., 2011, *N* = 16–20; Simcock and Dooley, 2007, *N* = 20; Tare et al., 2010, *N* = 18). Eleven additional participants were tested but excluded because of experimenter error (child was too young, *N* = 1; experimenter did not adhere to testing protocol, *N* = 5; experimenter accidentally tested child multiple times under different IDs, *N* = 3; experimenter ran child in wrong counterbalance condition, *N* = 2), three additional participants were tested but excluded because of non-compliance, and pre-test data were collected from 30 additional participants but were not included because participants were either absent or unavailable for the storybook reading and post-test when we returned to the school a week later.

#### Materials

**FIGURE 2:**
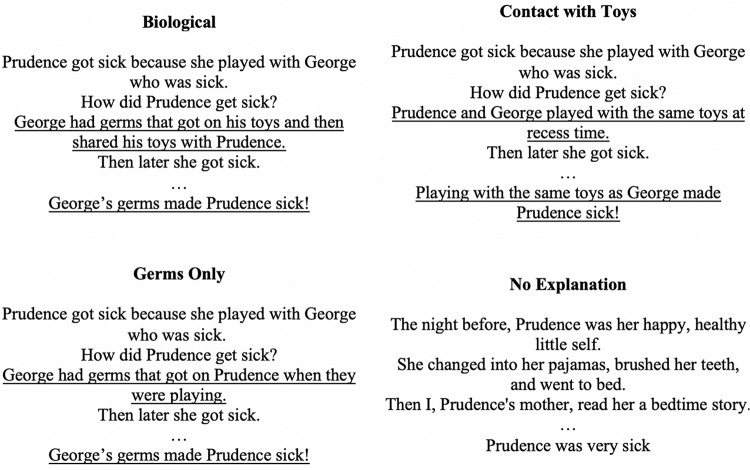
Explanatory text from the four storybooks created for Study 2.

Excerpts from a commercial storybook, *Prudence’s Get Well Book* (Frankel, 2000), were used as the stimuli. The story was about a little girl, Prudence, who became ill with a fever. Her mother took her to the doctor and gave her medicine, and later, Prudence returned to school well again. Three different contagion-relevant versions of the book were created, each providing different information about how Prudence got sick by changing only one page (see Figure 2 for relevant text). All three storybooks said that Prudence got sick after an interaction with a friend, George. The only thing that differed between the books was the specific information about how Prudence got sick from playing with George. In the *biological storybook*, children were told, “George had germs that got on his toys and then shared his toys with Prudence. Then later she got sick.” In the *germs-only storybook*, children were told, “George had germs that got on Prudence when they were playing. Then later she got sick.” In the *contact-with-toys storybook*, children were told, “Prudence and George played with the same toys at recess time. Then later she got sick.” We included the two latter conditions in order to account for any individual effects of providing children with information about germs and information about sharing objects that are contaminated. In addition to the three contagion-relevant storybook conditions, we had two control conditions: a *no-storybook* condition, where participants were simply given both the pre- and post-test assessments with no book reading, and a *no-explanation storybook* condition, in which children were read the same story, except without any causal explanation for how Prudence may have gotten sick.

#### Procedure

Children sat with the experimenter in a quiet area of the classroom or separate testing room and were tested individually at both pre-test and post-test. Children were told that they were going to look at some pictures and/or read a storybook and that we were going to ask them some questions about it. The procedure involved a verbal knowledge pre-test, storybook intervention, verbal knowledge post-test, and behavioral avoidance task. The verbal knowledge post-test and behavioral avoidance task occurred directly after the storybook intervention, which was conducted approximately 1 week after the verbal knowledge pre-test. We chose to do the storybook intervention a week after the pre-test to avoid asking children the same questions repeatedly in a single session and to avoid taxing the preschools we visited with multiple visits in the same week. Children in the no-book control condition completed the verbal knowledge and behavioral avoidance tasks at both pre-test and post-test.

##### Pre-test

At pre-test, we assessed (1) children’s knowledge of contagious and non-contagious illness with an explanation and prediction task and (2) their germ knowledge. Given that knowledge of illness transmission may vary based on socio-economic factors, schooling, and culture, we used these pre-test assessments from the three book conditions to establish that children did not differ in their baseline knowledge. In particular, one of our book conditions included information about germs, so we asked questions about children’s germ knowledge specifically to ensure that germ knowledge at pre-test did not differ between conditions.

There is currently no widely used assessment of children’s knowledge of illness transmission. Previous research suggests that preschool-aged children are able to answer simple forced-choice, yes-versus-no questions about illness transmission accurately. For example, as mentioned above, when given a choice between two explanations for the cause of a cold, preschool-aged children are more likely to endorse contagion-relevant explanations (e.g., from playing with a friend who had a cold) than immanent justice explanations (i.e., getting sick as a punishment for misbehaving; Siegal, 1988; Springer and Ruckel, 1992). However, when asked open-ended interview questions, children of the same age also overextend contagion to non-contagious events, such as accidents (scraped knee) and non-contagious illnesses (toothache), and endorse non-physical causes of illness transmission that include immanent justice (Kister and Patterson, 1980). Thus, we asked a combination of forced-choice yes/no questions along with open-ended questions to ensure that we obtained results consistent with previous research.

###### Illness vignettes

During the first portion of the pre-test, the children were read vignettes about a child with a cold and a child with a toothache. For the cold vignette, they were shown a picture of a child and were told: “This is Sal. Sal has a cold, so Sal has a runny nose, a headache, and sore throat.” For the toothache vignette, they were shown a picture of a different child and were told: “This is Danny. Danny has a toothache, so his tooth really hurts when he tries to eat or drink anything.” The children were then prompted to provide an open-ended explanation for how the child got a cold or toothache: “How did Sal/Danny get a cold/toothache?” Afterward, they were asked to make predictions about whether they and another child would get a cold or toothache after playing with the child in the vignette: “Do you think Sal/Danny’s friend could get a cold/toothache by playing with Sal/Danny? Do you think Sal/Danny’s friend could get a cold/toothache by being mean to Sal/Danny? What if you played with Sal/Danny? Would you get a cold/toothache?” The order of presentation of the cold and toothache questions was counterbalanced across participants.

Children’s open-ended explanation answers (“How did Sal/Danny get a cold/toothache?”) for both pre- and post-testing were coded using the same categories as storybook coding in Study 1: contagion-relevant explanations (biological, physical proximity) and contagion-irrelevant explanations (behavioral, non-physical, unspecific/not relevant). However, children’s explanations were generally very short, and there were no cases where children’s responses contained more than one category. Thus, the categories were treated as mutually exclusive. Children’s responses to each of the prediction questions about person-to-person transmission of colds and toothaches were coded as 1 for a correct response (for each of the cold questions: yes, no, yes, respectively; for toothache questions: no, no, no, respectively) and as 0 for an incorrect response (for each of the cold questions: no, yes, no, respectively; for toothache questions: yes, yes, yes, respectively). The first author coded all of the transcripts, blind to storybook condition. A second coder (undergraduate research assistant) independently coded 42% of the transcripts to establish interrater reliability. Percent agreement between the coders was 86%, and the Cohen’s kappa was 0.79, which is considered a moderate level of agreement. Any disagreements were resolved by discussion, and a final code was agreed upon by both coders.

###### Germ knowledge measure

Because one of our book conditions provides children with information about germs, we assessed children’s germ knowledge at pre-test to ensure that there were no differences between children in each condition at baseline. Again, we used both open-ended and forced-choice questions. First, children were asked to tell the experimenter everything they knew about germs. Answers were coded based on providing no information or irrelevant information (“they’re tiny”), behavioral knowledge related to germs (“mommy told me you have to wash your hands”), or understanding germs as a cause of illness (“when we get germs on us we get colds”). If children provided both behavioral knowledge and causal explanations in their responses, the answer was coded as the latter, reflecting a more sophisticated level of understanding. Next, a series of yes/no questions were asked. Children were asked whether sick people have germs (correct = yes), whether people who aren’t sick have germs (correct = yes), and whether germs can make you sick (correct = yes). For the open-ended questions, the first author coded all of the transcripts blind to storybook condition. A second coder (undergraduate research assistant) independently coded 74% of the transcripts to establish interrater reliability. Percent agreement between the coders was 74%, and Cohen’s kappa was 0.63, which is considered a moderate level of agreement. Any disagreements were resolved by discussion, and a final code was agreed upon by both coders.

##### Storybook intervention and post-test

Approximately 1 week after the pre-test, an experimenter read the storybook to each child individually in a quiet area of the classroom or a separate room. Again, we chose to do the storybook intervention 1 week after the pre-test to avoid asking children the same questions repeatedly in a single session and to avoid taxing the preschools we visited with multiple visits in the same week. After reading the storybook, children were first asked three questions to probe their memory about the book: (1) what was the little girl’s name, (2) do you remember how Prudence got sick, and (3), what did Prudence do to get better? It is important to note that children’s accurate responses to these questions were low: only 8% of children accurately recalled Prudence’s name, 28% remembered how she got sick, and 59% remembered what she did to get better. After the memory questions, children were again administered the Illness Vignettes (with different names and photographs) to examine if children’s knowledge changed from before the intervention. The order of presentation of the cold and toothache questions was again counterbalanced across participants.

In addition, at post-test, a behavioral choice test was administered. The behavioral choice test measured children’s avoidance of objects that a child with a contagious illness had previously come in contact with. Children were told that the two children from the Illness Vignettes – the one with a cold and the one with a toothache – had played with two toys earlier. The children were then told that they could pick one to take home with them as a prize for reading the book. The children were then presented with two identical toys and asked whether they wanted the one that the child with the toothache had played with or the one that the child with the cold had played with. Children’s toy choices were scored as 0 (cold) or 1 (toothache).

### Results

#### Data Analysis Plan

All of the data we collected were binary (yes/no) or categorical in nature, which is common of similar paradigms (e.g., Bonawitz et al., 2012). Thus, in the following analyses, to test for differences on outcome variables between the book conditions (between-subjects), we used chi-square analyses. To test for differences on outcome variables between pre- and post-test (within-subjects), we used McNemar Exact Tests. Finally, to test for differences from chance, we used binomial tests.

#### Baseline Knowledge

First we investigated children’s knowledge at pre-test to ensure that there were no differences at baseline between the book conditions and to provide simple descriptive data of preschool-aged children’s knowledge of illness transmission. Chi-square analyses revealed that there were no initial differences at pre-test across storybook conditions on any of the questions, *p*’s > 0.05. According to a series of binomial tests (Table 1), at pre-test, most children reported that germs can make you sick (78%) and that sick people have germs (86%); further, they said that a friend could catch a cold by playing with a child who has a cold (68%), *p*’s < 0.001. However, they did not apply this knowledge to themselves and were at chance when asked whether playing with someone who has a cold could make them sick as well (48%), *p* = 0.78. Children were at chance when asked whether non-sick people have germs, when asked whether a child could get a cold by being mean to another child, and for all of the questions about toothaches except for one (*p*’s > 0.05); in response to whether a friend could get a toothache by playing with another child who had a toothache, they answered with “yes” or “I don’t know” at a rate that was greater than chance (77%), *p* = 0.002. This suggests that the children tested were not necessarily sure about whether being mean to someone resulted in getting sick or a toothache and whether a toothache is contagious.

**TABLE 1:** Children’s performance on closed-ended pre-test questions in the germ knowledge measure and illness vignettes across all conditions.

**Baseline measure**	**Question**	**Children who answered correctly**N** (%)**			
		**Contagion-relevant conditions (*N* = 71)**	**Control conditions (*N* = 48)**	**All conditions together (**N** = 119)**	****P**-value**
Germ knowledge measure	Do sick people have germs?	64 (90%)	38 (79%)	102 (86%)	< 0.001*
	Do people who aren’t sick have germs?	31 (44%)	28 (58%)	59 (50%)	1.000
	Can germs make you sick?	56 (79%)	31 (76%)	87 (78%)	< 0.001*
Illness vignette – cold	Do you think Sal/Danny’s friend could get a cold by playing with Sal/Danny?	48 (68%)	33 (69%)	81 (68%)	< 0.001*
	Do you think Sal/Danny’s friend could get a cold by being mean to Sal/Danny?	34 (48%)	22 (46%)	56 (47%)	0.582
	Would you get a cold if you played with Sal/Danny?	29 (41%)	28 (58%)	57 (48%)	0.783
Illness vignette – toothache	Do you think Sal/Danny’s friend could get a toothache by playing with Sal/Danny?	26 (37%)	16 (33%)	42 (35%)	0.002*
	Do you think Sal/Danny’s friend could get a toothache by being mean to Sal/Danny?	38 (54%)	16 (33%)	64 (54%)	0.463
	Would you get a toothache if you played with Sal/Danny?	39 (56%)	19 (40%)	58 (49%)	0.927

*P*-values from binomial test for differences from chance. *Signifies a *p*-value < 0.05.

Although children showed some competence in answering the yes/no questions correctly for a contagious illness, they were unable to give contagion-relevant explanations for a cold in the open-ended question (Table 2). Only a small number of children gave contagion-relevant explanations for a cold: 4% gave an explanation involving physical proximity, while only 2% mentioned germs. The majority of children gave either a behavioral explanation (38%) or no explanation (56%) for why someone would get sick.

**TABLE 2:** Coding categories of children’s provided open-ended explanations at baseline for the questions “How did Sal/Danny get a cold/toothache?” across all conditions.

**Explanation category**	**How did Sal/Danny get a cold? *N* (%)**			**How did Sal/Danny get a toothache? *N* (%)**		
	**Contagion-relevant conditions (*N* = 71)**	**Control conditions (*N* = 48)**	**All conditions together (*N* = 119)**	**Contagion-relevant conditions (*N* = 71)**	**Control conditions (*N* = 48)**	**All conditions together (*N* = 119)**
Biological	2 (3%)	0 (0%)	2 (2%)	1 (1%)	0 (0%)	1 (1%)
Physical Proximity	3 (4%)	2 (4%)	5 (4%)	2 (3%)	0 (0%)	2 (2%)
Behavioral	26 (37%)	19 (40%)	45 (38%)	33 (46%)	24 (50%)	57 (48%)
Non-physical	0 (0%)	0 (0%)	0 (0%)	0 (0%)	0 (0%)	0 (0%)
Unspecific/not relevant	40 (56%)	27 (56%)	67 (56%)	35 (49%)	24 (50%)	59 (50%)

#### Post-test Knowledge

Post-test knowledge was used to assess the primary question of what children learned from the storybook conditions from pre-test to post-test. There were no significant differences between the three contagion conditions at post-test for any of the questions, nor were there significant differences at post-test between the two control conditions, so data were collapsed within these two categories (contagion-relevant versus control).

There were no significant differences from pre-test to post-test on any of the test questions for the control conditions (Table 3). However, children provided significantly more accurate, contagion-relevant explanations from pre-test to post-test in the contagion-relevant conditions (*p* = 0.012). Further, they also showed significant improvement from pre-test to post-test when asked whether they could get a cold by playing with another child who has a cold (*p* = 0.011). More specifically, at pre-test, only 41% of children in the contagion-relevant book conditions said you could get a cold by playing with another child who had a cold, while 62% answered this question correctly after the storybook interaction. Likewise, only 7% (5 total children) gave a correct, contagion-relevant explanation for how a child might get sick at pre-test, while 20% (14 total children) provided an accurate contagion-relevant explanation to this question at post-test, after reading the storybook.

**TABLE 3:** Pre- to post-test comparisons for cold knowledge across the contagion-relevant and control conditions.

	**Contagion-relevant conditions**			**Control conditions**		
	**Pre-test *N* (%)**	**Post-test *N* (%)**	***P*-value**	**Pre-test *N* (%)**	**Post-test *N* (%)**	***P*-value**
How did Sal/Danny get a cold? Contagion-relevant explanations.	5 (7%)	14 (20%)	0.012*	2 (4%)	6 (13%)	0.219
Would you get a cold if you played with Sal/Danny?	29 (41%)	44 (62%)	0.011*	28 (58%)	25 (52%)	0.508

*P*-values from within-subjects McNemar Exact Tests. *Signifies a *p*-value < 0.05.

#### Behavioral Post-test

**FIGURE 3:**
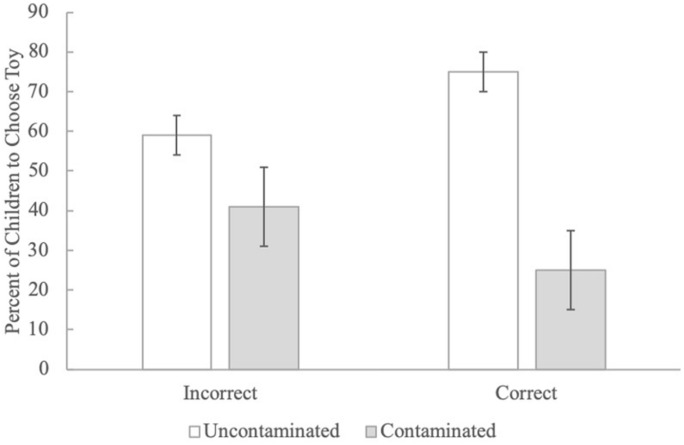
The percent of children who chose the uncontaminated toy in the behavioral post-test across conditions based on whether they answered the open-ended contagion question correctly at post-test.

Our final question was whether children who learned from the book interventions were also more likely to choose the uncontaminated object. Thus, we focused on children’s behavioral choice based on the answers to the two questions (open-ended contagion question/Can you get sick by playing with another sick child?) that garnered significant improvement at post-test. Unfortunately, overall learning was low – for example, only a handful of children (14 in the contagion condition and 6 in the control conditions) answered the open-ended post-test question correctly – so we did not have the statistical power to examine differences in behavioral choice between conditions. However, across conditions, children who answered the open-ended question correctly at post-test across conditions chose the uncontaminated toy at a level that was above hance, *p* = 0.041 (Figure 3). Children who answered the open-ended question incorrectly at post-test chose between the two toys at chance, *p* > 0.05. There were no significant differences for behavioral choice based on whether or not they thought they could get a cold by playing with another child who had a cold at post-test, *p*’s > 0.05.

### Discussion

The results of Study 2 demonstrate that children who were read a storybook with contagion-relevant information about illness transmission showed significant improvement in their knowledge about illness transmission from pre-test to post-test. Second, behavioral choice was related to knowledge: children who answered the open-ended contagion question correctly at post-test chose the uncontaminated toy significantly more often than children who answered this question incorrectly.

## General Discussion

Although previous research has focused heavily on the development of children’s contagion knowledge (e.g., Kalish, 1996; Keil et al., 1999), we still know very little about the content of children’s daily experiences that might be relevant to knowledge development and, most importantly, how this knowledge translates to adaptive behavior. The current research focused on examining whether storybook reading – a common daily experience that has been shown to contribute to the development of biological knowledge (e.g., Williams and Binnie, 2002; Myant and Williams, 2008; Gonzalez et al., 2011) – can potentially impact children’s knowledge about contagion. Most notably, the current study is the very first to explore how their knowledge is related to behavior when presented with a contaminated object; there is no previous research that examines whether highlighting contagion-relevant causal information will encourage children to actively avoid germs in a similar situation. Studying whether children can learn biological information from storybooks that leads to health-promoting behaviors might be particularly important for developing age-appropriate explanations for children experiencing illness (Koopman et al., 2004) and for creating health education interventions to improve children’s knowledge and subsequent behavior when faced with the risk of infection (Williams and Binnie, 2002; Myant and Williams, 2008).

Here, we found that children’s commercial storybooks rarely contained biological causal information about illness. Instead, many children’s stories about illness focus on the physical and emotional consequences of being sick and sometimes present physical proximity as an important factor in predicting contagion. Thus, storybooks do not seem to be a source from which children can typically gather causal information about illness transmission. In our second study, we asked about the role that storybooks – specifically, storybooks that contain explanations for illness transmission that were mostly absent from the commercial books – play in the development of children’s knowledge and behavior. We found that after providing preschool-aged children with a book containing one of several causal explanations about illness transmission, children were more likely to use contagion-relevant explanations for how someone might get sick than before the book reading. Most importantly, children who provided these accurate, contagion-relevant explanations at post-test were more likely to avoid a contaminated toy than children who did not accurately provide these explanations.

These results are novel and important for two reasons. First, although we already knew from previous research that storybooks can be used to teach children biological knowledge, our data suggest that a very simple storybook manipulation can lead to increases in knowledge about illness transmission in children as young as preschool age. Taken together with the results of Study 1, our findings suggest that while most children’s storybooks do not provide causal information about illness transmission, storybooks about contagion with relevant causal information can be used to teach children about illness transmission. Second, this is the very first study demonstrating that providing children with causally relevant information about illness transmission in a simple storybook is related to adaptive health behaviors.

Despite these novel contributions, the current work also has some important limitations. While our age range was generally in line with similar storybook learning and contagion knowledge research, we did not have a large-enough sample size to explore age-related differences. However, previous research has reported that knowledge, and not age, is the best predictor of children’s illness avoidance behavior; indeed, even the youngest children in Blacker and LoBue’s (2016) sample avoided a sick confederate if they had causal knowledge about illness transmission (Blacker and LoBue, 2016). However, further exploring the developmental trajectory across early childhood is an important goal for future research. Further, although children’s knowledge did improve in the contagion-relevant conditions, very few children learned overall, with only 20 children answering the open-ended contagion question correctly at post-test. Given that most children answered the yes/no contagion questions correctly at pre-test, these findings suggest that learning about illness might happen in a piecemeal fashion, consistent with what other researchers have proposed (e.g., Legare et al., 2009). Despite the small number of children who learned from our storybooks, children who answered the open-ended contagion question correctly at post-test consistently tended to choose the uncontaminated toy. The very small number of children in each cell made us unable to analyze the individual book conditions separately, limiting our ability to make specific claims about what types of knowledge lead to healthy behaviors. Although we cannot make claims about which exact type of information might be most effective, the small number of children who learned across conditions suggests that stronger manipulations are likely needed for long-term knowledge gain and behavioral change in future examinations.

Perhaps the limited learning we observed should not be surprising: the storybook intervention we used was very simple, each book contained very little information about the cause of illness transmission, and books were only read to children once. Further, children had trouble remembering details of the story, as evidenced by very low rates of accurate responding to questions probing details about the story (8–59% accuracy). We found the most gains with the most stringent outcome variable possible to measure learning, namely, children’s ability to generate correct contagion-relevant explanations about how someone might get sick (as opposed to responses to yes/no or forced-choice questions). It is possible that more children were unable to answer this question correctly simply because of limitations in their verbal communication skills. In the current study, we did not include a measure of either receptive or expressive language, and it is possible that individual differences in language skills played a role in whether children were able to generate contagion-relevant explanations. Thus, although we cannot draw specific conclusions about the type of information most relevant for knowledge and behavioral change, our data do suggest that preschool-aged children might be able to learn about illness transmission from a book interaction and apply their knowledge to behave adaptively. Additionally, whether our results reflect lasting knowledge and behavioral change is unknown. Longitudinal research in this area would certainly be beneficial to better understanding the long-term impact of a brief storybook intervention on children’s knowledge and behavior. Despite the fact that our findings were relatively weak, this work constitutes only an exploratory first attempt at addressing this important issue, and our results open the door to future work that provides children with more extensive information about transmission and administers book readings at more than one time point so that children might improve more than they did by our very simple manipulation. Multiple storybooks that address different aspects of contagion may be required to truly elicit complex and coherent explanations in preschoolers.

Importantly, our results also suggest that information found in children’s everyday storybooks does not necessarily provide them with accurate information about illness transmission. This leaves open the question of *where* and *how* children acquire knowledge about illness transmission from their everyday experiences. It is possible that parents provide such information, or that children receive this information from school programs, but this area of research is still largely understudied. Further, it is not clear how children learn to generalize their knowledge to other contagious illness and when they learn *not* to generalize to non-contagious illness, like a toothache. Indeed, a sophisticated understanding of illness transmission would include an understanding of how to apply knowledge to contagious illnesses and how to generalize that knowledge when appropriate. Likewise, a sophisticated understanding of illness transmission would allow children to make predictions about how illness exposure would affect themselves as well as others. Interestingly, while children were able to correctly indicate that contact with a sick person could make a friend sick, they failed to answer this question correctly for themselves at pre-test. It is likely that in the preschool years, children are still developing these abilities, which is why responding for these questions might have been inconsistent. Future work is still needed to identify the types of everyday experiences that lead to children’s knowledge revision and eventually to a sophisticated understanding of illness transmission.

In conclusion, given the recent widespread concern over the spread of contagious illnesses among children, including the flu and the measles, ensuring that children have the necessary information to avoid potentially contagious individuals and contaminated objects is especially important. Unfortunately, because of the dearth of research on children’s behavior in this domain, especially in preschool-aged children, current interventions aimed to teach children health-related behaviors such as hand-washing and sanitizer use in the classroom have focused only on older children. Further, most interventions are structured primarily around preventative behaviors while providing little information about *why* these behaviors reduce illness transmission (see Au et al., 2008, for a review; Witta and Spencer, 2004; Colgate-Palmolive Company, 2010). In fact, knowledge interventions and behavioral interventions have largely been separate bodies of work, and no studies have examined whether causal information about illness transmission influences health-related behavior, such as active avoidance of germs. And while previous interventions have often focused on teaching children behavioral information (such as hand-washing), it is not clear whether behavioral interventions alone lead to illness prevention (e.g., Rosen et al., 2006). Our results suggest that interventions that incorporate biological causal information may be a brand-new avenue for teaching children healthy behaviors so that they can play a role in their own disease avoidance.

## Data Availability Statement

The data sets generated for this study can be found in the Databrary repository, upon request to the corresponding author.

## Ethics Statement

The studies involving human participants were reviewed and approved by the Institutional Review Board Rutgers University – Newark. Written informed consent to participate in this study was provided by the participants’ legal guardian/next of kin.

## Author Contributions

MC, K-AB, and VL contributed to the conception and design of the study. EK and ZW oversaw the data collection. MC and VL performed the statistical analysis and completed the first draft of writing. MC, K-AB, VL, and EK contributed to the manuscript revision.

## Conflict of Interest

The authors declare that the research was conducted in the absence of any commercial or financial relationships that could be construed as a potential conflict of interest.
