# Supplementary material for: Using Storybooks to Teach Children About Illness Transmission and Promote Adaptive Health Behavior – A Pilot Study
Source: Front Psychol. 2020 Jun 5;11:942. doi: 10.3389/fpsyg.2020.00942 (PMC7289927; doi:10.3389/fpsyg.2020.00942)
Supplement: Supplementary file 1 [file Table_1.DOCX]

Supplementary Material

## Appendix A: Children’s Books (Title, Author, Publication Year) Analyzed in Study 1

A River Dream by Allen Say (1988)

A Sick Day for Amos McGee by Philip Stead (2010)

A Visit from Dr. Katz by Ursula Le Guin (1988)

Angelina and the Princess by Katharine Holabird (1984)

Barnyard Song by Rhonda Gowler Greene (1997)

Bear Feels Sick by Karma Wilson (2007)

Brianna, Jamaica, and the Dance of Spring by Juanita Havill (2002)

Brundibar by Tony Kushner (2004)

Carl and the Sick Puppy by Alexandra Day (2012)

Carousel by Brian Wildsmith (1988)

Chicken Soup by Heart by Esther Hershenhorn (2010)

Don't You Feel Well, Sam? by Amy Hest and Anita Jeram (2002)

Dr. Duck by H. M. Ehrlich and Laura Rader (2000)

Feel Better, Ernest! by Gabriel Vincent (1988)

Felix Feels Better by Rosemary Wells (2001)

Germs Make Me Sick by Melvin Berger (1985)

Get Well, Clown-Arounds by Joanna Cole (1982)

Get Well Soon, Adam by Sarah, Duchess of York (2011)

Get Well, Good Knight by Shelley Moore Thomas (2002)

Goldie Locks has Chicken Pox by Erin Dealey (2002)

Guess Who, Baby Duck! By Amy Hest (2004)

Henry and Nudge Get the Cold Shivers by Cynthia Rylant (1989)

How Do Dinosaurs Get Well Soon? by Jane Yolen and Mark Teague (2003)

I Wish I Was Sick, Too! by Franz Brandenberg and Aliki (1990)

I'm Not Feeling Well Today by Shirley Neitzel (2001)

I've Got Chicken Pox by True Kelley (1994)

If Beaver had a Fever by Helen Ketteman (2011)

I'm Really Ever So Not Well by Lauren Child (2007)

Iris and Walter: The School Play by Elissa Haden Guest (2000)

Katie Woo Has the Flu by Fran Manushkin (2011)

Llama Llama Home with Mama by Anna Dewdney (2011)

Look by Michael Grejniec (1993)

Madeline by Ludwig Bemelmans (1939)

Mama Provi and the Pot of Rice by Sylvia Rosa-Casanova (1997)

Marsha Makes Me Sick by Barbara Bottner (1998)

Mary Alice, Operator Number 9 by Jeffrey Allen (1975)

Miss Malarkey Won't Be in Today by Judy Finchler (1998)

Monkey and Elephant Get Better by Carole Lexa Schaefer (2013)

Monkey Soup by Louis Sachar (1992)

Mother Mother I Feel Sick, Send for the Doctor, Quick Quick Quick by Remy Charlip (1966)

Mr. Putter & Tabby Catch the Cold by Cynthia Rylant (1994)

My Cold Went on Vacation by Molly Rausch (2011)

Nora's Roses by Satomi Ichikawa (1993)

One Cow Coughs by Christine Loomis (1994)

Rooster Can't Cock-a-Doodle-Doo by Karen Rostoker-Gruber (2004)

The Berenstain Bears: Sick Days by Jan Berenstain and Mike Berenstain (2009)

So Sick! By Harriet Ziefert (1985)

Taking Care of Mama by Mitra Modarressi (2010)

Taking Care of Mama Rabbit by Anita Lobel (2014)

Tashi and the Tibetan Flower Cure by Naomi Rose (2011)

Teddy Bears Cure a Cold by Susanna Gretz (1984)

The Berenstain Bears Go to the Doctor by Jan Berenstain and Mike Berenstain (1981)

The Best Cat Suit of All by Sylvia Cassedy (1991)

The Big Little Sneeze by Katja Reider (2002)

The Germ Busters by Rosemary Wells (2002)

The Ice Palace by Angela McAllister (1994)

The Island Light by Rosemary Wells (1992)

The Octopus by Denys Cazet (1990)

The Princess of Borscht by Leda Schubert (2011)

The Sick Bug by Susie Bazil (2008)

The Sniffles for Bear by Bonny Becker and Kady MacDonald Denton (2011)

The Winter Day by Beverly Komoda (1991)

Tomato Soup by Thacher Hurd (1992)

What the Mailman Brought by Carolyn Craven (1987)

When Daddy Had the Chicken Pox by Harriet Ziefert (1991)

When Francie Was Sick by Holly Keller (1985)

When Vera Was Sick by Vera Rosenberry (1998)
